# Research on group type theory and its functorial semantic models in category logic

**DOI:** 10.1371/journal.pone.0326301

**Published:** 2025-06-24

**Authors:** Jian-Gang Tang, Yimamujiang Aishan, Ji-Yu Liu, Jia-Yin Peng

**Affiliations:** 1 Division of Mathematics, Sichuan University Jinjiang College, Meishan, China; 2 College of Mathematics and Statistics, Kashi University, Kashi, Xinjiang, China; 3 College of Mathematics and Statistics, Yili Normal University, Yining, Xinjiang, China; 4 Civil Aviation Logistics Technology Co., Ltd., Chengdu, Sichuan, China; Minnan Normal University, CHINA

## Abstract

This paper explores the introduction of group structures within type theory, drawing from the algebraic theory proposed by Roy L. Crole. We define types with group structures and demonstrate that models of these types in categories with finite products can be interpreted as group objects. Each equation within the context of group theory types corresponds to a commutative diagram, representing the axioms of groups, inspired by Lawvere’s functorial semantics. Moreover, we clarify the role of control equations associated with fundamental properties of groups, such as operations and identities. By formalizing a type referred to as “Group Type," which involves integrating group operations and the equations they satisfy into an algebraic type, we incorporate the algebraic structure of a specific group into this type. This Group Type represents a concrete algebraic structure. In practical applications, the introduction of group structures into types is anticipated to optimize algorithms and data structures, leveraging the algebraic properties of groups to enhance computational efficiency. Furthermore, our exploration is not limited to mathematical conversions; it is also envisioned to extend to the application in various type systems, providing support for future research in formal verification and program analysis in computer science.

## Introduction

Type theory was first introduced by Russell and Church, who emphasized the necessity of types to resolve logical paradoxes [2,3]. In 1940, Church developed a framework for simple type theory, laying the groundwork for subsequent research [1]. This theory has become a key tool for understanding computation and logic, providing a theoretical foundation for addressing logical challenges.

In modern logic, type theory based on category theory is evolving into a new unified foundation for contemporary mathematics. Categorical logic originated from Lawvere’s Functorial Semantics of Algebraic Theories [4] and the Elementary Theory of the Category of Sets [5]. Broadly speaking, categorical logic represents syntax and semantics through categories, while interpretations are conveyed through functors. In the realm of theoretical computer science, categorical logic and type theory find a wide range of applications in functional programming languages, program semantics, and program logic.

As an extension and development of category theory, mathematical logic, and programming, categorical logic and type theory have seen rapid advancements in recent years. Lawvere [6] provided insights into the relationship between logic and layers. Makkai and Reyes [7] introduced and studied first-order categorical logic. Lambek and Scott [8] made significant contributions by introducing higher-dimensional categorical logic. Andrew M. Pitts [19] further developed the theory of categorical logic. Bart Jacobs [20] systematically summarized the research achievements in the domains of categorical logic and type theory. Lastly, John Baez and Mike Stay [22] explored connections among physics, topology, logic, and computation within the framework of category theory.

Roy L. Crole’s book [9] explores the relationship between category theory, logic, and type theory, focusing on several key aspects. He provides a detailed introduction to the foundational concepts of category theory, including objects, morphisms, and the relationships between categories, as well as their applications in logic and computational theory. Additionally, he examines logical systems from a categorical perspective, analyzing how different logical systems can be described using categorical language. Crole also investigates the structure of type theory, emphasizing its significance in programming languages and computer science. He discusses the interplay between logic, type theory, and category theory, illustrating how these fields influence and enhance one another. Furthermore, he offers multiple examples that demonstrate the application of these theories in the design and analysis of actual programming languages and systems. Overall, Crole’s research provides important insights into the profound connections among these domains, holding significant implications for both academic research and practical applications.

With the development of programming languages, type theory’s applications in computer science have become increasingly widespread. Milner proposed a theory of type polymorphism, establishing the foundation for functional programming languages [10]. Pierce’s book “Types and Programming Languages" systematically summarizes various concepts of type systems [11].

In program development, interactive theorem proving tools such as Coq and Lean have become important research directions. Bertot and Cast$\overset{´}{e}$ran’s book provides a detailed introduction to using Coq for program development [12]. The Lean user guide offers practical tools and resources for users [13].

In recent years, research on the semantics of probabilistic programming languages has gradually emerged. Kozen explored the semantics and reasoning of probabilistic programs in his works [14,15]. Jia and colleagues proposed research on commutative monads for probabilistic programming languages, filling a gap in this field [16].

In the context of quantum computing, Jia and colleagues researched the semantics of variational quantum programming, advancing the theoretical development of quantum programming languages [17]. Additionally, Goubault-Larrecq and colleagues proposed a domain-theoretic approach to explore issues related to statistical programming languages [18].

The applications of type theory and categorical logic in programming languages, as well as the research advances in probabilistic and quantum programming, have greatly enriched the theoretical framework and provided new tools and ideas for practical applications. This opens up new perspectives for us to further explore the intersection and integration of these theories and their applications in emerging technologies.

The theoretical foundation of programming languages is rooted in categorical logic. In this paper, we approach the introduction of group structures within the type system from a syntactic perspective, specifically defining types with group structures based on the algebraic theory proposed by Roy L. Crole. From a semantic standpoint, we demonstrate that the models of group structure types in categories with finite products can be interpreted as group objects. Within the context of group theory types, each equation corresponds to a commutative diagram associated with the group object in the semantic category, and each commutative diagram represents an axiom of the group. This approach also draws inspiration from Lawvere’s concept of functorial semantics of categories. Specifically, the model in the category of sets corresponds to a classical group, while the model in n-dimensional manifolds is associated with Lie groups.

When referencing control equations in the context of group theory types, it is important to clarify the name, meaning, and role of each equation within the theory. Examples of control equations and their citation methods typically involve fundamental properties of groups, such as group operations, commutativity, identity elements, and inverse elements.

The type system provides a formal mathematical foundation for expressing and managing complex structures. By introducing group structures in type theory, we can more accurately model the basic properties of groups, including group operations, identity elements, and inverse elements. By defining a type called ‘Group‘, which includes the elements of the group, operations, and verification of related properties, we can ensure that the group structures used in program design conform to mathematical definitions while also enhancing the clarity and maintainability of the code. Furthermore, directly integrating the properties of groups into the definition of types helps to automatically verify group characteristics in programs, ensuring compliance with group theory requirements.

In program design, the introduction of group structures can assist in solving many complex problems. For instance, many algorithms and data structures (such as hash tables and cryptographic algorithms) can be optimized by leveraging the properties of groups. By applying group structures in design, we can utilize the symmetry and algebraic properties of groups to enhance computational efficiency, resulting in more effective and reliable solutions.

In terms of theoretical groundwork, researching how to introduce group structures in types is not limited to the conversion of mathematical formulas but also necessitates exploring applications in different types of systems (such as dependent type systems and modal type systems). This in-depth theoretical research will lay the foundation for the application of group theory in computer science, such as formal verification and program analysis.

## Preliminaries

Currently, various formulations of type theory can be found in the literature. This paper adopts a simplified version of the algebraic type theory proposed by Roy L. Crole [9]. In this algebraic type theory, we introduce operations and equations with a group structure, which is one of the distinctive features of this work, this algebraic type theory is referred to as “Group Type Theory." This approach can also be applied to the study of more complex algebraic type theories.

The first step is to introduce a simplified version of the algebraic type theory concept proposed by Roy L. Crole [9].

Definition2.1 [9] An algebraic type theory 𝔸=(Sg,Ax) consists of the following components:

where *Sg* includes:

(1) Types: σ,τ,.....;

(2) Function symbols: f,g,......, where $f:\sigma_{1},\sigma_{2},......\sigma_{n}\to \tau$, when *n* is zero, *f* is called a constant function symbol, and is denoted as k:τ;

(3) The formation rules for initial terms:

$\begin{array}{c} \overset{―}{x} \end{array}$ $\begin{array}{c} \overset{―}{k} \end{array}$ $\begin{array}{c} M_{1}\;\cdots \;M_{a}\\ \overset{―}{f(M_{1},\cdots ,M_{a})} \end{array}$

In other words:

1. A variable is a term;

2. A constant is a term;

3. If $M_{1},...,M_{a}$ are terms, then $f(M_{1},...,M_{a})$ is a term, where $f:\sigma_{1},\sigma_{2},......\sigma_{a}\to \tau$.

(4) The formation rules for proven terms:

$\begin{array}{c} \overset{―}{\Gamma ,x:\sigma ,\Gamma^{\prime }⊢x:\sigma } \end{array}$ $\begin{array}{c} \overset{―}{\Gamma ⊢k:\sigma } \end{array}(k:\sigma )$

$\begin{array}{c} \underset{―}{\Gamma ⊢M_{1}:\sigma_{1}\;...\;\Gamma ⊢M_{a}:\sigma_{a}}\\ \Gamma ⊢f(M_{1},...,M_{a}):\tau \end{array}(f:\sigma_{1},...,\sigma_{a}\to \tau )$

Among them, Γ is called the context, usually denoted as $\Gamma =[x_{1}:\sigma_{1},...,x_{n}:\sigma_{n}]$, where $x_{1},...,x_{n}$ are different variables, and $\sigma_{1},...,\sigma_{n}$ are types. A form like Γ⊢M:σ is called a term under the context, where *M* is the original term. Given *Sg*, a proven term is a term under the context generated by the above rules and is denoted as Sg▷Γ⊢M:σ.

The class of equations under the context about *Sg* is denoted as *Ax*, where an equation under the context has the form $\Gamma ⊢M=M^{\prime }:\sigma$, here, Γ⊢M:σ and $\Gamma ⊢M^{\prime }:\sigma$ are proven terms. An equation under the context in *Ax* is called an axiom of this theory and is denoted as $Ax▷\Gamma ⊢M=M^{\prime }:\sigma$.

(5) The theorems of 𝔸=(Sg,Ax) (having the form $\Gamma ⊢M=M^{\prime }:\sigma$) are produced by the following rules:

$\begin{array}{c} \underset{―}{Ax▷\Gamma ⊢M=M^{\prime }:\sigma }\\ \Gamma ⊢M=M^{\prime }:\sigma \end{array}$ $\begin{array}{c} \underset{―}{Sg▷\Gamma ⊢M:\sigma }\\ \Gamma ⊢M=M:\sigma \end{array}$ $\begin{array}{c} \underset{―}{\Gamma ⊢M=M^{\prime }:\sigma }\\ \Gamma ⊢M^{\prime }=M:\sigma \end{array}$

$\begin{array}{c} \underset{―}{\Gamma ⊢M=M^{\prime }:\sigma \;\Gamma ⊢M^{\prime }=M^{\prime \prime }:\sigma }\\ \Gamma ⊢M=M^{\prime \prime }:\sigma \end{array}$ $\begin{array}{c} \Gamma ⊢M=M^{\prime }:\sigma \\ \overset{―}{\pi \Gamma ⊢M=M^{\prime }:\sigma } \end{array}$ (where π is a permutation)

$\begin{array}{c} \Gamma ⊢M=M^{\prime }:\sigma \\ \overset{―}{\Gamma^{\prime }⊢M=M^{\prime }:\sigma } \end{array}$ (where $\Gamma \subseteq \Gamma^{\prime }$)

$\begin{array}{c} \underset{―}{\Gamma ,x:\sigma ⊢N=N^{\prime }:\tau \;\Gamma ⊢M=M^{\prime }:\sigma }\\ \Gamma ⊢N\;\;[\;\;M/x\;]=N^{\prime }\;\;[\;\;M^{\prime }/x\;]:\tau \end{array}$

This paper now introduces equations with a group structure based on a simplified algebraic type theory proposed by Roy L. Crole, which we refer to as “group type theory."

**Definition 2.2** A group type theory 𝔾 has:

(1) A type σ;

(2) Function symbols: i:σ→σ, m:σ,σ→σ, e:σ;

(3) The formation rules for initial terms:

$\begin{array}{c} \overset{―}{x} \end{array}$ $\begin{array}{c} \overset{―}{e} \end{array}$ $\begin{array}{c} M_{1}\;M_{2}\\ \overset{―}{m(M_{1},M_{2})} \end{array}$ $\begin{array}{c} M\\ \overset{―}{i(M)} \end{array}$

(4) The rules for generating proven terms:

$\begin{array}{c} \overset{―}{\Gamma ,x:\sigma ,\Gamma^{\prime }⊢x:\sigma } \end{array}$ $\begin{array}{c} \overset{―}{\Gamma ⊢e:\sigma } \end{array}(e:\sigma )$ $\begin{array}{c} \underset{―}{\Gamma ⊢M_{1}:\sigma \;\Gamma ⊢M_{2}:\sigma }\\ \Gamma ⊢m(M_{1},M_{2}):\sigma \end{array}$ $\begin{array}{c} \Gamma ⊢M:\sigma \\ \overset{―}{\Gamma ⊢i(M):\sigma } \end{array}$

(5) The equations under the context, which are axioms, are:

$\begin{array}{c} \underset{―}{\Gamma ⊢x:\sigma \;\Gamma ⊢e:\sigma }\\ \Gamma ⊢m(e,x)=x:\sigma \end{array}(a)$ $\begin{array}{c} \underset{―}{\Gamma ⊢x:\sigma \;\Gamma ⊢e:\sigma }\\ \Gamma ⊢m(x,e)=x:\sigma \end{array}(b)$

$\begin{array}{c} \Gamma ⊢x:\sigma \\ \overset{―}{\Gamma ⊢m(x,i(x))=e:\sigma } \end{array}(c)$ $\begin{array}{c} \Gamma ⊢x:\sigma \\ \overset{―}{\Gamma ⊢m(i(x),x)=e:\sigma } \end{array}(d)$

$\begin{array}{c} \Gamma ⊢x:\sigma \;\Gamma ⊢y:\sigma \;\Gamma ⊢z:\sigma \\ \overset{―}{\Gamma ⊢m(x,m(y,z))=m(m(x,y),z):\sigma } \end{array}(e)$

Theorem generation rules are consistent with the generation rules of algebraic type theory.

## Functorial semantic models of group type theory

In this section, we will introduce functorial semantic models of group type theory. To this end, we first present the concept of an interpretation (or structure) of a general algebraic type theory in a category with finite products, as defined by Roy L. Crole. In this paper, we only require the categories related to functorial semantics to have finite products, whereas in the study of more complex type theories, the categories involved in functorial semantics typically need to have Cartesian closedness.

**Definition 3.1** [9] Let 𝔸=(Sg,Ax) be an algebraic type theory and 𝒞 be a category with finite products. An interpretation (or structure) *M* of *Sg* in 𝒞, satisfies:

(1) The types σ in *Sg* are objects [[σ]] in 𝒞;

(2) The function symbols $f:\sigma_{1},\sigma_{2},......\sigma_{n}\to \tau$ in *Sg* are morphisms $\left[\;[f]\;]:[\;[\sigma_{1}]\;]\times [\;[\sigma_{2}]\;]\times ...\times [\;[\sigma_{n}]\;]\to [\;[\tau ]\;\right]$ in 𝒞;

(3) The constant function symbols k:σ in *Sg* are morphisms [[k]]:1→[[σ]] in 𝒞, where 1 is the terminal object in 𝒞.

**Note 3.1** For each proven term of the algebraic type theory 𝔸=(Sg,Ax), we define [[Γ⊢M:σ]]:[[Γ]]→[[σ]] in 𝒞 as a morphism, where $\Gamma =[x_{1}:\sigma_{1},...,x_{n}:\sigma_{n}]$ is the context and $[\;[\Gamma ]\;]\overset{def}{=}[\;[\sigma_{1}]\;]\times ...\times [\;[\sigma_{n}]\;].$ Thus, we obtain:


$$\begin{array}{c} \overset{―}{\left[\;\left[\Gamma ,x:\sigma ,\Gamma^{\prime }⊢x:\sigma \right]\;\right]\overset{def}{=}\;\pi :\left[\;\left[\Gamma \right]\;\right]\times \left[\;\left[\sigma \right]\;\right]\times \left[\;\left[\Gamma^{\prime }\right]\;\right]\to \left[\;\left[\sigma \right]\;\right]} \end{array}$$

$$\begin{array}{c} \overset{―}{\left[\;\left[\Gamma ⊢k:\sigma \right]\;\right]\overset{def}{=}\;\left[\;\left[k\right]\;\right]\circ !:\left[\;\left[\Gamma \right]\;\right]\to 1\to \left[\;\left[\sigma \right]\;\right]} \end{array}(k:\sigma )$$

$\begin{array}{c} \left[\;\left[\Gamma ⊢M_{1}:\sigma_{1}\right]\;\right]=m_{1}:\left[\;\left[\Gamma \right]\;\right]\to \left[\;\left[\sigma_{1}\right]\;\right]\;\ldots \;\left[\;\left[\Gamma ⊢M_{n}:\sigma_{n}\right]\;\right]=m_{n}:\left[\;\left[\Gamma \right]\;\right]\to \left[\;\left[\sigma_{n}\right]\;\right]\\ \overset{―}{\left[\;\left[\Gamma ⊢f(M_{1},\ldots ,M_{n}):\tau \right]\;\right]=\left[\;\left[f\right]\;\right]\circ <m_{1},\ldots ,m_{n}>:\left[\;\left[\Gamma \right]\;\right]\to \left[\;\left[\sigma_{1}\right]\;\right]\times \ldots \times \left[\;\left[\sigma_{n}\right]\;\right]\to \left[\;\left[\tau \right]\;\right]} \end{array}$

We now introduce the concept of a model of a general algebraic type theory in a category with finite products, as defined by Roy L. Crole.

**Definition 3.2.** [9] Let 𝔸=(Sg,Ax) be an algebraic type theory and 𝒞 a category with finite products. An interpretation **M** of *Sg* in 𝒞, for an equation $\Gamma ⊢M=M^{\prime }:sigma$ under the context, if **M** makes [[Γ⊢M:σ]] and $\left[\;\left[\Gamma ⊢M^{\prime }:\sigma \right]\;\right]$ equal morphisms in 𝒞, then we say that **M** satisfies this equation under the context. If **M** satisfies all equations under the context in *Ax*, then we call **M** a model of the algebraic type theory 𝔸=(Sg,Ax).

We will now introduce the concept of group objects in categories with finite products.

**Fig 1:**
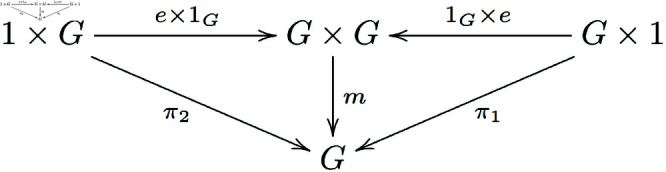
In a general category with finite products, morphisms of group objects that satisfy the existence of an identity element form a commutative diagram.

**Fig 2:**
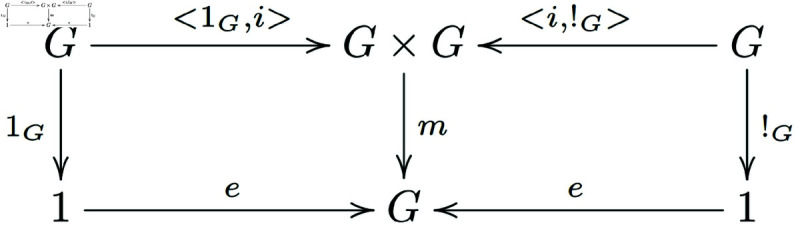
In a general category with finite products, morphisms that satisfy the existence of inverses for group objects form a commutative diagram.

**Fig 3:**
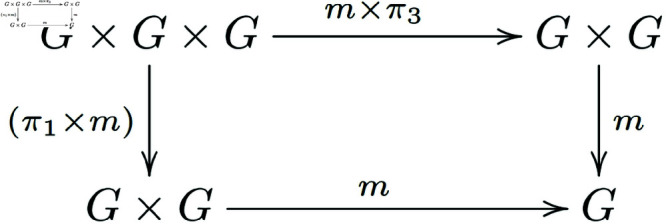
In a general category with finite products, morphisms that satisfy the associativity condition for group objects form a commutative diagram.

Definition3.3. [21] Let 𝒞 be a category with finite products, and let G∈ob(𝒞), e:1→G, m:G×G→G, i:G→G be morphisms such that the following diagrams commute (Fig 1):

Existence of a unit *e*:

Existence of inverses (Fig 2):

Associativity of *m* (Fig 3):

Then (G,m,i,e) is called a group object in the category 𝒞.

**Note 3.2** The provided definitions are describing a group object in a category 𝒞 with finite products. The first diagram represents the associativity of the multiplication, the second one shows the existence of a unit (or identity element), and the third one demonstrates the existence of inverses for each element in *G*.

**Lemma 3.1.** [9] The structure of a group type theory 𝔾 in a category 𝒞 with finite products is as follows: types are objects in the category 𝒞; proven items are morphisms between objects in 𝒞.

Specifically:


$$\begin{array}{c} \overset{―}{\left[\;\left[\Gamma ,x:\sigma ,\Gamma^{\prime }⊢x:\sigma \right]\;\right]\overset{def}{=}\;\pi :\left[\;\left[\Gamma \right]\;\right]\times \left[\;\left[\sigma \right]\;\right]\times \left[\;\left[\Gamma^{\prime }\right]\;\right]\to \left[\;\left[\sigma \right]\;\right]} \end{array}$$

$$\begin{array}{c} \overset{―}{\left[\;\left[\Gamma ⊢e:\sigma \right]\;\right]\overset{def}{=}\;\left[\;\left[e\right]\;\right]\circ !:\left[\;\left[\Gamma \right]\;\right]\to 1\to \left[\;\left[\sigma \right]\;\right]} \end{array}(e:\sigma )$$

$$\begin{array}{c} \left[\;\left[\Gamma ⊢M_{1}:\sigma \right]\;\right]=m_{1}:\left[\;\left[\Gamma \right]\;\right]\to \left[\;\left[\sigma \right]\;\right]\;\left[\;\left[\Gamma ⊢M_{2}:\sigma \right]\;\right]=m_{2}:\left[\;\left[\Gamma \right]\;\right]\to \left[\;\left[\sigma \right]\;\right]\\ \overset{―}{\left[\;\left[\Gamma ⊢m(M_{1},M_{2}):\sigma \right]\;\right]=\left[\;\left[m\right]\;\right]\circ <m_{1},m_{2}>:\left[\;\left[\Gamma \right]\;\right]\to \left[\;\left[\sigma \right]\;\right]\times \left[\;\left[\sigma \right]\;\right]\to \left[\;\left[\sigma \right]\;\right]} \end{array}(m:\sigma ,\sigma \to \sigma )$$

$$\begin{array}{c} \left[\;\left[\Gamma ⊢N:\sigma \right]\;\right]=n:\left[\;\left[\Gamma \right]\;\right]\to \left[\;\left[\sigma \right]\;\right]\\ \overset{―}{\left[\;\left[\Gamma ⊢i(N):\sigma \right]\;\right]:\left[\;\left[i\right]\;\right]\circ n:\left[\;\left[\Gamma \right]\;\right]\to \left[\;\left[\sigma \right]\;\right]\to \left[\;\left[\sigma \right]\;\right]} \end{array}(i:\sigma \to \sigma )$$

**Note 3.3** For convenience in writing, we will abbreviate [[Γ]]asT, [[σ]]asG, [[m]]asm, and [[i]]asi.

We will now discuss models of group type theory in a general category with finite products.

**Theorem 3.1** A model of the group type theory 𝔾 in the category 𝒞 with finite products is a group object in the category 𝒞.

**Proof.** We will begin with equations (a) and (b) from Definition 2.2 and, based on the interpretations of types, function symbols, and equations as provided in Definitions 3.1 and 3.2, demonstrate the validity of the commutative diagram 1 stated in Definition 3.3.

**Fig 4:**
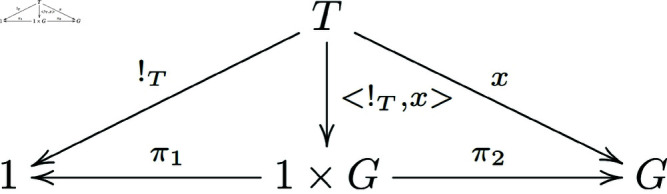
The commutative diagram satisfied by the morphisms in the proof of Theorem 3.1.

From the interpretation of the proven items and axiom (a), we know that $m\circ <e\circ {!}_{T},x>=x$,

As shown in the following diagram (Fig 4):

By the universal property of the product, we have $\pi_{1}\circ <{!}_{T},x>={!}_{T}$ and $\pi_{2}\circ <{!}_{T},x>=x$.

Therefore, we obtain $m\circ <e\circ {!}_{T},x>=\pi_{2}\circ <{!}_{T},x>=x$.

**Fig 5:**
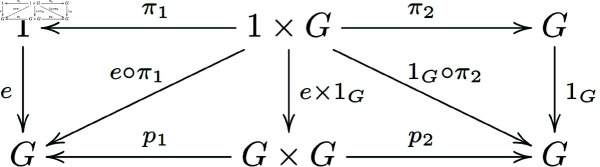
The commutative diagram satisfied by the morphisms in the proof of Theorem 3.1.

In the following diagram (Fig 5):

By the universal property of the product, we know that $<e\circ \pi_{1},1_{G}\circ \pi_{2}>=e\;\times \;1_{G}$.

**Fig 6:**
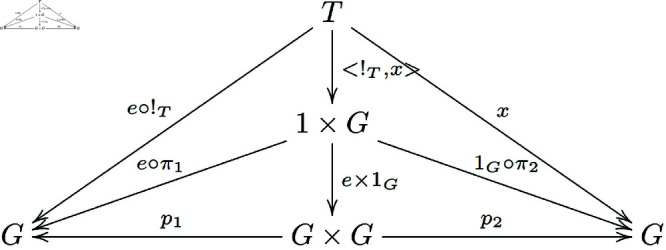
The commutative diagram satisfied by the morphisms in the proof of Theorem 3.1.

Because $(e\circ \pi_{1})\circ <{!}_{T},x>=e\circ (\pi_{1}\circ <{!}_{T},x>)=e\circ {!}_{T}$ and similarly, $(1_{G}\circ \pi_{2})\circ <{!}_{T},x>=1_{G}\circ (\pi_{2}\circ <{!}_{T},x>)=1_{G}\circ x=x$. Thus, all small triangles in the diagram below exchange (Fig 6).

Hence, $p_{1}\circ (e\;\times \;1_{G})\circ <{!}_{T},x>=(p_{1}\circ (e\;\times \;1_{G}))\circ <{!}_{T},x>=(e\circ \pi_{1})\circ <{!}_{T},x>=e\circ {!}_{T}$. Similarly, $p_{2}\circ (e\;\times \;1_{G})\circ <{!}_{T},x>=(p_{2}\circ (e\;\times \;1_{G}))\circ <{!}_{T},x>=(1_{G}\circ \pi_{2})\circ <{!}_{T},x>=x$.

**Fig 7:**
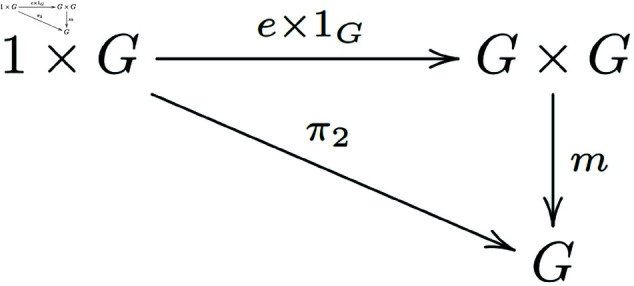
In a general category with finite products, the morphisms of group objects that satisfy the existence of a left identity element form a commutative diagram.

**Fig 8:**
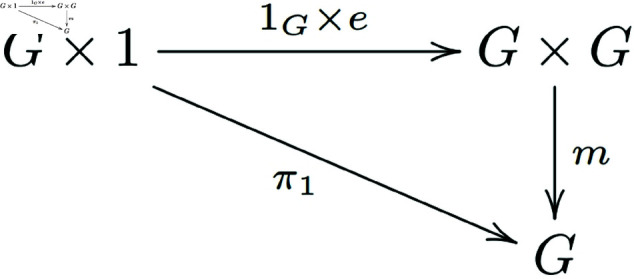
In a general category with finite products, the morphisms of group objects that satisfy the existence of a right identity element form a commutative diagram.

From the above discussion, we know that $m\circ <e\circ {!}_{T},x>=m\circ ((e\;\times \;1_{G})\circ <{!}_{T},x>)=(m\circ (e\;\times \;1_{G}))\circ <{!}_{T},x>=\pi_{2}\circ <{!}_{T},x>$, then $m\circ (e\;\times \;1_{G})={pi}_{2}$. That is, the diagram below exchanges (Fig 7):

Similarly, we can prove that the diagram below also exchanges (Fig 8):

That is $m\circ (1_{G}\;\times \;e)=\pi_{1}$.

Secondly, we will begin with equations (c) and (d) from Definition 2.2 and prove the validity of commutative diagram 2 as stated in Definition 3.3.

**Fig 9:**
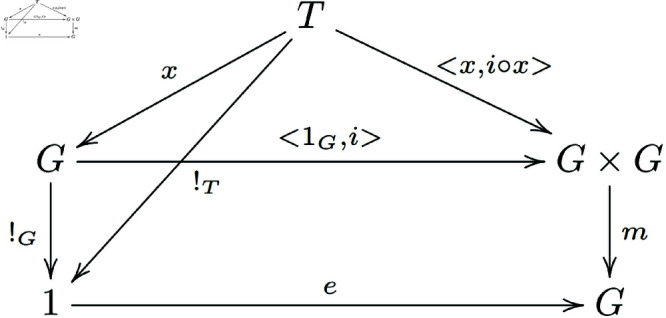
The commutative diagram satisfied by the morphisms in the proof of Theorem 3.1.

By the interpretation of the proven items and axiom (c), we know that $m\circ <x,i\circ x>=e\circ {!}_{T}$ (Fig 9).

**Fig 10:**
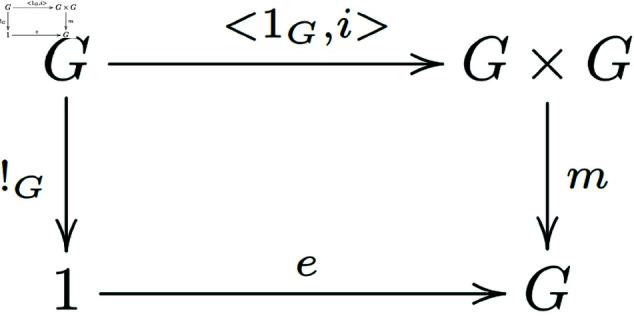
Morphisms of group objects that satisfy the existence of a right inverse element form a commutative diagram.

**Fig 11:**
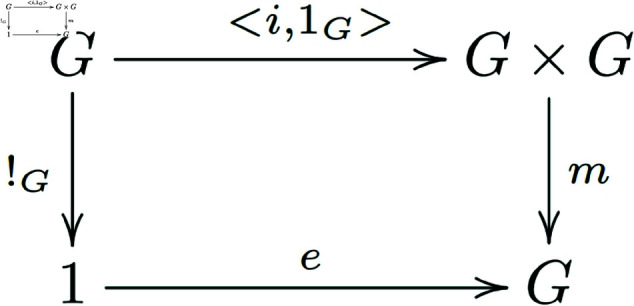
Morphisms of group objects that satisfy the existence of a left inverse element form a commutative diagram.

Because $<1_{G},i>\circ x=<x,i\circ x>$, and because 1 is a terminal object, we can get ${!}_{G}\circ x={!}_{T}$, then $(m\circ <1_{G},i>)\circ x=m\circ (<1_{G},i>\circ x)=m\circ <x,i\circ x>=e\circ {!}_{T}=e\circ ({!}_{G}\circ x)=\text{(}e\circ {!}_{G})\circ x$, then we can get $m\circ <1_{G},i>=e\circ {!}_{G}$. That is, the diagram below exchanges (Fig 10):

Similarly, the diagram below also exchanges (Fig 11):

Finally, we will start with equation (e) from Definition 2.2 and demonstrate the validity of commutative diagram 3 as described in Definition 3.3.

By the interpretation of the proven items and axiom (e), we know that m∘<x, m∘<y,z>>=m∘<m∘<x,y>,z>, $(m\;\times \;\pi_{3})\circ <x,y,z>=<m\circ <x,y>,z>$, $(\pi_{1}\;\times \;m)\circ <x,y$, z>=<x,m∘<y,z>>, where <x,y,z>:T→G×G×G. Then $m\circ (m\;\times \;\pi_{3})\circ <x,y,z>=m\circ <m\circ <x,y>,z>=m\circ <x,m\circ <y,z>>=m\;\circ \;(\pi_{1}\;\times \;m)\circ <x,y,z>$, then we can get $m\;\circ \;(m\;\times \;\pi_{3})=m\circ (\pi_{1}\;\times \;m)$.

**Fig 12:**
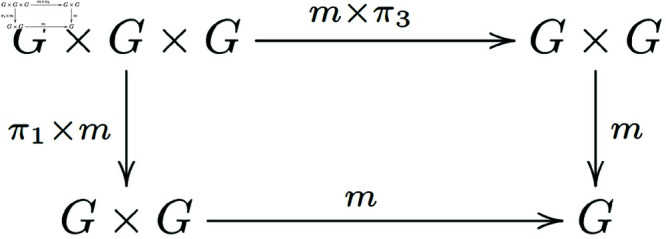
Morphisms of group objects that satisfy the associativity condition form a commutative diagram.

That is, the diagram below exchanges (Fig 12):

In summary, the Functorial semantic model of the group type theory 𝔾 in a category 𝒞 with finite products satisfies all the conditions for a group object as stated in Definition 3.3. Therefore, the Functorial semantic model of the group type theory 𝔾 is a group object in the category 𝒞.

## Models of the group type theory 𝔾 in concrete categories

**Theorem 4.1.** Let 𝔾 be the group type theory. A model of the group type theory in the category of sets Set is a standard group G=(G,m,i,e), where G∈ob(𝐂), and the mappings e:1→G, m:G×G→G, and i:G→G satisfy the following properties:

1. *Closure:* For all x,y∈G, the product m(x,y)=x·y∈G.
2. *Associativity:* For all x,y,z∈G, it holds that m(m(x,y),z)=m(x,m(y,z)).
3. *Existence of Identity:* There exists an element e∈G such that for all x∈G, m(e,x)=x and m(x,e)=x.
4. *Invertibility:* For each x∈G, there exists an element y∈G denoted as i(x)) such that m(x,y)=e and m(y,x)=e.

Thus, the structure (G,m,e,i) forms a group under the operation defined by *m*.

We will explain in detail that the group type theory 𝔾 has a model in the category of sets Set, which is a group object in the category Set.

**Proof.** For convenience in notation, we will abbreviate [[Γ]]asT, [[σ]]asG, [[m]]asm, and [[i]]asi. Since *T* is a singleton set, we can assume without loss of generality that *T* = {*e*}. The definition in the model category Set can be interpreted as follows:

(1) A type σ can be interpreted as a non– empty set *G*.

(2) The function symbols i:σ→σ, m:σ×σ→σ, and e:σ can be interpreted as mappings e:1→G, m:G×G→G, and i:G→G, representing the constant mapping, inverse mapping, and product mapping, respectively.

(3) The formation rules for initial terms are expressed as follows:

$\begin{array}{c} \overset{―}{x} \end{array}$ $\begin{array}{c} \overset{―}{e} \end{array}$ $\begin{array}{c} M_{1}\;M_{2}\\ \overset{―}{m(M_{1},M_{2})} \end{array}$ $\begin{array}{c} M\\ \overset{―}{i(M)} \end{array}$

These can be interpreted in the model category Set as follows: the variable *x* belongs to *G*, the constant *e* belongs to *G*; if the terms *M*_1_ and *M*_2_ belong to *G*, then the term $m(M_{1},M_{2})$ also belongs to *G*, and i(M) likewise belongs to *G*.

(4) The rules for generating proven terms are given by:

$\begin{array}{c} \overset{―}{\Gamma ,x:\sigma ,\Gamma^{\prime }⊢x:\sigma } \end{array}$ $\begin{array}{c} \overset{―}{\Gamma ⊢e:\sigma } \end{array}(e:\sigma )$ $\begin{array}{c} \underset{―}{\Gamma ⊢M_{1}:\sigma \;\Gamma ⊢M_{2}:\sigma }\\ \Gamma ⊢m(M_{1},M_{2}):\sigma \end{array}$ $\begin{array}{c} \Gamma ⊢M:\sigma \\ \overset{―}{\Gamma ⊢i(M):\sigma } \end{array}$

These can be interpreted in Set as follows: the first rule states that if *x* has type σ, then it is an element of *G*. The second rule states that *e* has type σ, meaning e∈G. The third rule indicates that if *M*_1_ and *M*_2_ have type σ, then the term $m(M_{1},M_{2})$ also has type σ, thus $m(M_{1},M_{2})\in G$. The fourth rule states that if *M* has type σ, then i(M) has type σ as well, which implies i(M)∈G.

(5) The axioms under the context are as follows:

$\begin{array}{c} \underset{―}{\Gamma ⊢x:\sigma \;\Gamma ⊢e:\sigma }\\ \Gamma ⊢m(e,x)=x:\sigma \end{array}\;(a)$ $\begin{array}{c} \underset{―}{\Gamma ⊢x:\sigma \;\Gamma ⊢e:\sigma }\\ \Gamma ⊢m(x,e)=x:\sigma \end{array}\;(b)$

$\begin{array}{c} \Gamma ⊢x:\sigma \\ \overset{―}{\Gamma ⊢m(x,i(x))=e:\sigma } \end{array}\;(c)$ $\begin{array}{c} \Gamma ⊢x:\sigma \\ \overset{―}{\Gamma ⊢m(i(x),x)=e:\sigma } \end{array}\;(d)$

$\begin{array}{c} \Gamma ⊢x:\sigma \;\Gamma ⊢y:\sigma \;\Gamma ⊢z:\sigma \\ \overset{―}{\Gamma ⊢m(x,m(y,z))=m(m(x,y),z):\sigma } \end{array}\;(e)$

The axioms (equations) can be interpreted as follows:

(a) This axiom states that for any element *x* of type *sigma* and the identity element *e*, the operation m(e,x) yields *x*, thus demonstrating the left identity property.

(b) Similarly, this axiom asserts that for any element *x* of type *sigma* and the identity element *e*, the operation m(x,e) also yields *x*, illustrating the right identity property.

(c) This axiom indicates that for any element *x* of type σ, the result of the operation m(x,i(x)) equals *e*, which shows that i(x) is indeed the inverse of *x*.

(d) This axiom shows that for any element *x* of type σ, the operation m(i(x),x) also equals *e*, confirming that i(x) serves as the inverse of *x*.

(e) Finally, this axiom states that for any elements *x*, *y*, and *z* of type σ, the operation m(x,m(y,z)) is equal to m(m(x,y),z), thereby demonstrating the associativity of the operation *m*.

Thus, G=(G,m,i,e) satisfies the three commutative diagrams specified in Definition 3.3, indicating that G=(G,m,i,e) is a group object in the category of sets Set. Therefore, the interpretation of group theory 𝔾 in this context is a group object in the category of sets Set.

**Definition 4.1** Let *G* be a group equipped with a topology. If the topology on *G* renders the group multiplication operation f:G×G→G,(x,y)↦xy and the inversion operation $i:G\to G,x\mapsto x^{-1}$ continuous, where G×G carries the product topology, then *G* is called a topological group.

**Theorem 4.2** The category of topological spaces Top models the group theory 𝔾 as topological groups.

*Proof:* By Theorem 3.1, the model of the group theory 𝔾 in the category of topological spaces Top is the group object (G,m,i,e) in Top, which ensures that *i* and *m* are continuous functions. Moreover, the definition of the group object (G,m,i,e) sustains the multiplication operation m:G×G→G,(x,y)↦xy and the inversion operation $i:G\to G,x\mapsto x^{-1}$. Therefore, the group theory 𝔾 in the category of topological spaces Top models as topological groups.

**Definition 4.2** [13] Let (G,·) be a group, and let ≤ be a partial order on *G*. For any a,b,g∈G, if a≤b implies both ag≤bg and ga≤gb, then (G,·,≤) is called an ordered group.

**Theorem 4.3** The group theory 𝔾 in the category of partially ordered sets (G,·,≤) is an ordered group.

*Proof:* By Theorem 3.1, we know that the model of group theory 𝔾 in the category of partially ordered sets Pos is the group object (G,m,i,e) within Pos, which implies that *i* and *m* are monotone mappings. Moreover, from the definition of the group object (G,m,i,e), *G* is a group such that for any a,b,g∈G, if a≤b, then m(a,g)≤m(b,g), and thus ag≤bg and ga≤gb. Therefore, the group theory 𝔾 in the category of partially ordered sets (G,m,i,e) is an ordered group.

**Definition 4.3** Let *X* be a topological space, and let $F:\Gamma (X){\;}^{OP}\to Set$ be a functor. If *F* satisfies that for any open set U∈Γ(X) and any open cover $U={⋃}_{i}U_{i}$, i∈I, such that the following diagram is an equalizer diagram, then *F* is called a sheaf on the topological space *X*.



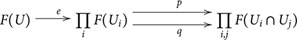



Here, $e:t\mapsto (F(U_{i}\subseteq U)(t))$, $p:(t_{i})\mapsto (F(U_{i}\cap U_{j}\subseteq U_{i})(t_{i}))$, and $q:(t_{i})\mapsto (F(U_{i}\cap U_{j}\subseteq U_{j})(t_{j}))$.

Note: For any two sheaves *F* and *G* on a topological space *X*, a morphism between *F* and *G* is a natural transformation α:F→G. Hence, we can form the full subcategory Sh(X) of the functor category $\left[\Gamma (X){\;}^{op},\text{Set}\right]$, whose objects are all sheaves on the topological space *X*.

**Theorem 4.4** The model of group theory 𝔾 in the category of sheaves Sh(X) is a group sheaf.

*Proof:* By Theorem 3.1, the model of group theory 𝔾 in the category of sheaves Sh(X) is a group object (G,m,i,e) within Sh(X). It follows that *G* is a sheaf and satisfies the commutative diagrams as per Definition 3.3. Therefore, for any open set *U*, G(U) is a group and hence (G,m,i,e) constitutes a group sheaf. Consequently, the model of group theory 𝔾 in the category of sheaves Sh(X) is a group sheaf.

**Note 4.2** From the above theorem, we realize that using the model of group theory in categories with finite products, we can define concepts like topological groups, ordered groups, and group sheaves, thus unifying the concepts of topological groups, ordered groups, and group sheaves.

**Definition 4.4** A Lie group is a mathematical object that has both a smooth structure and a group structure. Specifically, a Lie group is defined as follows:

A Lie group *G* is a manifold that satisfies the following conditions:

1. Group Structure: There are two operations defined on *G*: - A multiplication map m:G×G→G such that $m(g_{1},g_{2})=g_{1}g_{2}$. - An inverse map i:G→G such that *i*(*g*) = *g*^−1^.

These operations satisfy the group axioms: associativity, identity element existence, and inverse element existence.

2. Smooth Structure: The multiplication map *m* and the inverse map *i* are both smooth functions (i.e., infinitely differentiable).

Thus, a Lie group is not only an algebraic structure but also a smooth manifold, allowing the use of differential geometry to study its properties.

**Theorem 4.5.** The models of group type theory in the category of *n*-dimensional smooth manifolds form a Lie group.

**Proof.** To show that the models of group type theory in the category of *n*-dimensional smooth manifolds form a Lie group, we need to verify two main properties:

1. Group Structure: We need to define the group operations on these models. Let *G* be the model of the group type. The group operation m:G×G→G can be defined via the composition of morphisms corresponding to the group operation in the theory. The identity element can be defined by an appropriate morphism in the category.

The inverse operation i:G→G is similarly defined through the morphisms that correspond to the inversion in the group theory.

2. Smooth Structure: Since *G* is a model in the category of *n*-dimensional smooth manifolds, we can endow *G* with a smooth structure. The operations *m* and *i* can be shown to be smooth. This follows from the fact that composition of smooth maps is smooth and the structure of the category ensures that the operations respect the smooth manifold structure.

Combining these two points, we conclude that *G* satisfies the properties of a Lie group. Therefore, the models of group type theory in the category of *n*-dimensional smooth manifolds indeed form a Lie group.

Finally, based on the above research, we can obtain the following deeper results.

We can define the notion of a topological group as a group object in CompHaus and a commutative Hopf C^*^– algebra as a group object in $ComUnC^{*}Algop$.

**Definition 4.5.** Let 𝒞 be a category with finite coproducts. We define,


$$\text{CoGrp}(𝒞):=\text{Grp}\;({𝒞}^{op}{)}^{\text{op}},$$

and call the objects of CoGrp (𝒞) cogroup objects in 𝒞 and the morphisms internal cogroup homomorphisms in 𝒞.

**Definition 4.6.** The objects of the category


compTopGrp:=Grp(compHaus)

are called compact topological groups. The objects of


$$\text{comHopf}\;C^{*}\text{-Alg}:=\text{CoGrp}(\text{comUn}C^{*}\text{-Alg})$$

are called commutative Hopf C^*^-algebras.

**Theorem 4.6.** There is an equivalence of categories,


$$\text{compTopGrp}=\text{Grp}(\text{compHaus})≃\text{Grp}\;\text{comUn}C^{*}{\text{-Alg}}^{\text{op}}=\text{comHopf}\;C^{*}{\text{-Alg}}^{\text{op}}$$

provided by the functors,


$$\text{Grp}\;C(\cdot ):\text{compTopGrp}\to \text{comHopf}\;C^{*}{\text{-Alg}}^{\text{op}}$$

and


$$\text{Grp}\;\sigma (\cdot ):\text{comHopf}\;C^{*}{\text{-Alg}}^{\text{op}}\to \text{compTopGrp}.$$

## Discussion

Type theory is profoundly connected to programming languages, particularly in the handling of variables, constants, and functions, where the underlying mathematical structures are often algebraic structures. Algebraic structures provide a theoretical foundation for computation, and group structures, as one of the most fundamental algebraic structures, offer powerful tools for understanding and analyzing various computational processes.

Integrating group structures into type theory, forming “Group Theory in Types,” represents an important and meaningful research direction. Group theory not only aids in understanding symmetry and structure but also provides a foundational framework for addressing many theoretical problems in computer science. By incorporating group structures into type theory, we can more effectively describe and analyze the various symmetries and transformations that occur during computation.

The different interpretations of group type theory reflect its applications within the framework of model categories:

In the category of sets Set, group objects can be viewed as standard sets, where the properties of group operations and identity elements are satisfied within the context of sets, providing an intuitive understanding of the fundamental properties of groups. In the category of topological spaces, group objects are interpreted as continuous groups, emphasizing the continuity of group operations within the topological structure, which allows us to study the geometric properties of groups and their applications in continuous transformations. In the category of *n*-manifolds, group objects can be understood as Lie groups, which are groups defined on smooth manifolds, particularly relevant for addressing differential structures, especially in the study of symmetries and conservation laws in physics. In the category of sheaves, group objects are viewed as group sheaves, enabling us to handle group structures at a more granular level, particularly in cases involving discontinuous or layered structures. In the category of groups, the interpretation of group objects as 2-groups extends the concept of groups, allowing us to explore the properties and behaviors of groups within higher categorical theories.

In summary, the introduction of group structures into type theory not only enriches the content of type theory but also fosters a deeper connection between computer science and algebraic structures. This interdisciplinary research enhances our understanding of computational processes and provides theoretical support for the design and implementation of programming languages.

In the research on programming language theory, the anticipated outcomes of integrating group theory and related algebraic structures are primarily reflected in several aspects:

1. **Type Safety and Program Verification:** By integrating the properties of group objects into type systems, we can enhance type safety mechanisms to effectively prevent type errors and verify algebraic properties during program execution, thereby improving the reliability and correctness of programs.
2. **Formal Verification and Model Checking:** Utilizing tools from group theory provides new perspectives for the formal verification of programs. By mapping program behaviors to group properties, we can develop effective model checking algorithms to automatically verify invariants and symmetries in programs, thus enhancing software quality.
3. **Symmetry in Concurrency and Distributed Computing:** Incorporating group structures in concurrent and distributed computing helps describe coordination and communication between computational nodes, simplifying system design and implementation while addressing consistency issues in distributed systems.
4. **Extension and Optimization of Type Systems:** Leveraging group structures to develop new type systems enhances the expressive power of languages and allows for optimizations at compile time, making the generated code more efficient, particularly in computations related to symmetry.
5. **Promotion of Interdisciplinary Research:** The integration of group theory with programming languages promotes interdisciplinary research between computer science, mathematics, and related fields, inspiring new research ideas and applications, and advancing areas such as quantum computing, cryptography, and artificial intelligence.

Through this research, the application of group theory within programming language theory can provide new tools and methods for scientific inquiry, driving innovation and progress in related fields.

## Conclusion

In this paper, we presented a comprehensive examination of the integration of group structures within type theory, highlighting both the syntactic and semantic dimensions of this endeavor. By defining types that encapsulate group structures based on the algebraic theory established by Roy L. Crole, we have established a formal framework that aligns closely with the principles of categorical logic. This approach allows for a robust representation of group properties, facilitating the accurate modeling of fundamental operations such as group multiplication, identity elements, and inverses.

We demonstrated that group structures in our proposed type system can be interpreted as group objects within categories possessing finite products, thereby enriching the semantic landscape of type theory. Each equation within the context of group theory types translates into a commutative diagram, reflecting the axiomatic nature of groups. This correspondence not only reinforces the logical coherence of the theory but also serves as a foundation for practical applications in programming languages, enhancing their expressiveness and reliability.

Furthermore, the incorporation of group structures into type systems offers significant advantages in program design and development. By leveraging the algebraic properties of groups, we can optimize algorithms and data structures, leading to more efficient computational solutions. This intersection of theoretical foundations and practical implementation underscores the potential of group theory to address complex challenges in computer science.

As we conclude, it is essential to recognize that the exploration of group structures in type theory is a stepping stone towards deeper investigations into various dependent and modal type systems. The theoretical insights gained from this research pave the way for further studies in formal verification, program analysis, and the design of advanced programming languages. Future work will focus on expanding these concepts and applying them to emerging technologies, thereby enriching the theoretical underpinnings of computation and fostering innovations in software development.

In summary, this research not only contributes to the understanding of the interplay between type theory and group theory but also lays the groundwork for future explorations of their applications in computer science, promising to enhance both academic inquiry and practical implementations in the field.
